# Biochemical and Physiological Changes during Early Adventitious Root Formation in *Chrysanthemum indicum* Linné Cuttings

**DOI:** 10.3390/plants11111440

**Published:** 2022-05-28

**Authors:** Bimal Kumar Ghimire, Seung-Hyun Kim, Chang-Yeon Yu, Ill-Min Chung

**Affiliations:** 1 Department of Crop Science, College of Sanghuh Life Science, Konkuk University, Seoul 05029, Korea; bimal_g12@yahoo.com (B.K.G.); kshkim@konkuk.ac.kr (S.-H.K.); 2 Bioherb Research Institute, Kangwon National University, Chuncheon 24341, Korea; cyyu@kangwon.ac.kr

**Keywords:** auxins, growth medium, temperature, explant type, adventitious root, *Chrysanthemum indicum*

## Abstract

*Chrysanthemum indicum* is an important ornamental and medicinal plant that is often difficult to propagate commercially because of its poor germination and low seed viability. This plant is mostly propagated by cutting, but the rooting is slow and non-uniform. The present investigation evaluated the regeneration capacity of stem cutting by examining the influence of auxins, growth medium, temperature, and explant type on adventitious root formation in *C. indicum*. The auxin-treated cuttings were planted in different growth substrates under greenhouse conditions. Among the different auxins tested, indole-3-butyric acid (IBA) more effectively induced roots. The cutting position of stock plants influenced rooting capacity. Cutting the stock plants from the apical region enhanced root number and length in the explants. Among the different explant types, apical stem cuts with 2000 ppm IBA produced a significantly higher number of adventitious roots when grown in vermiculite and perlite (V + P) at a ratio of 1:1 at 25 °C. High-performance liquid chromatography (HPLC) analysis revealed that protocatechuic acid, gentisic acid, chlorogenic acid, biochanin A, salicylic acid, caffeic acid, glycitein, and luteolin were the most dominant phenolic compounds present in *C. indicum*. These results indicate that IBA treatment promoted the synthesis and accumulation of phenolic compounds in *C. indicum* stem cuttings at the time of root formation. The present results demonstrate that applying auxins is essential for early root initiation and higher rooting success and thus may be beneficial for vegetative *C. indicum* propagation.

## 1. Introduction

*Chrysanthemum indicum* Linné (Asteraceae) is a perennial, aromatic, medicinal plant [1,2] distributed in Korea, Japan, China [3], Russia, and Europe [4]. Its flowers and buds are commonly used for traditional tea and to treat eye diseases in Korea and China [5] In addition to its medicinal value, due to its excellent aroma, these floral parts of the plants can also be added to rice cakes, as food additives for masking flavors, for making alcoholic beverages in Korea from ancient times [6]. The flowers bloom in September and October [1]. *C. indicum* extract is used as a traditional Chinese herbal medicine to lower blood pressure and treat nephritis, headaches [7,8], cancer, pneumonia, colitis, sores, fever, and stomatitis [9,10]. Extracts of this plant were shown to possess certain pharmacological properties, including inhibiting lens aldose reductase activity and nitric oxide production in lipopolysaccharide (LPS)-activated macrophages [2], and antimicrobial [11,12,13], antibacterial [14], anti-inflammatory [15,16], analgesic, antipyretic [11], and anticancer properties [2,5,17]. Furthermore, *C. indicum* has been shown to possess antioxidant properties [18,19] neuroprotective effects against oxidative stress [20], and hepatoprotective effects [21]. It also relieves hypertension and respiratory diseases [22,23].

One of the main problems encountered by propagation using seeds is the long dormancy, poor viability, and longer maturity attainment times [24]. Factors including the collection time, types of explants, temperature, plant size, stock plant age, and type of plant growth regulator (PGR) affect seed germination, causing late emergence or failure to germinate, reducing uniformity and yield [25,26,27,28]. Moreover, *Chrysanthemum* seeds have limited viability, short storage time, and a low germination rate. These problems can be overcome by clonal and mass propagation using superior genotypes. In contrast, stem cuttings are a principal alternative method for the propagation and production of high-quality plants [29]. It can maintain the progeny of elite plants with special features and qualities, resemble the mother plants in all respects [30], and are useful for quicker establishment and production [31,32].

It is a time-consuming and laborious process to establish the stock plant as the source of cuttings. Adventitious root formation is the key pre-requisite for successful establishment of cutting propagation. One of the main problems encountered by *C. indicum* during its clonal propagation is the low number of adventitious roots formed per cutting, negatively affecting plant growth and stability [33]. Exogenous auxin application not only helps initiate adventitious roots but also enhances the root numbers, improves root quality and uniformity, and reduces the time required for the rooting process [34,35,36]. Moreover, auxins are involved in most aspects of plant development [37], including main root formation and lateral and adventitious root initiation, by breaking cytokinin-induced root apical dominance [38]. Various studies have reported the advantages of applying exogenous rooting hormones and achieving early root emergence, enhanced root numbers and length, and improved quality and uniformity of rooted cuttings [39,40]. Auxins, such as indole-3-butyric acid (IBA), indole-3-acetic acid (IAA), and 1-Naphthaleneacetic acid (NAA), are used in a wide range of plants to promote root growth and induce the root system of cuttings [41]. Additionally, exogenous treatment with auxins reportedly increases the dry weight of roots [36], flower diameter [42], shoot length [43], leaf number [44], plant height, leaf area, and dry weight of shoots [45].

Polyphenols protect plants from oxidative stress [46] and UV light and prevent cell death from reactive oxygen species [47]. In addition, phenolic compounds act as auxin transport inhibitors [48,49]. Moreover, several previous studies have reported the influence of phenolic compounds on the rooting of cuttings by inhibiting auxin degradation [50,51]. Other studies have reported the important role of polyphenols in the rooting process of cuttings [52,53]. To our knowledge, the influence of auxins, temperature, and explant type on polyphenol content and *C. indicum* adventitious root formation have not been examined.

Therefore, the present study aimed to evaluate the regeneration potential of stem cuttings, establish the superior clonal stock of *C. indicum*, and identify the optimum auxin concentrations to enhance *C. indicum* rooting frequency. Likewise, this study compared the rooting response of cuttings at different temperatures and soil types and analyzed the effect of auxin treatment on the phenolic compounds in *C. indicum* cuttings and its relationship with rooting capacity.

## 2. Results

### 2.1. Effect of Auxins on Fresh Weight (FW) and Dry Weight (DW) of Roots and Shoots

**Figure 1:**
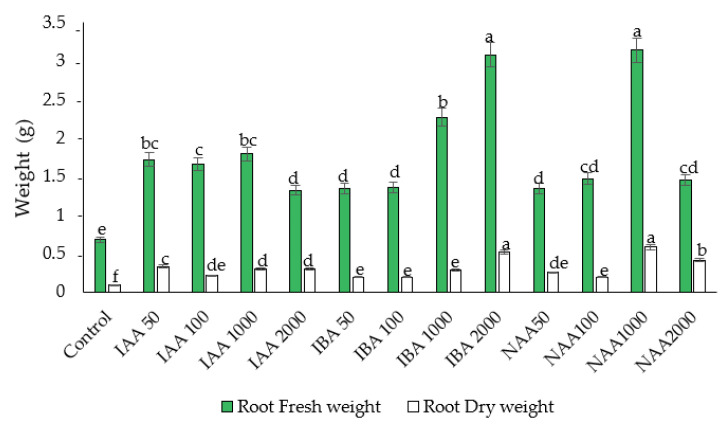
The effect of auxin concentration on root fresh and dry weight in *C. indicum.* Different letters indicate statistical differences as assessed by Duncan’s multiple comparison tests (*p* < 0.05).

All the auxin treatments significantly enhanced root FW and DW compared to the control. Except in some cases, all treatments showed an increasing trend in the FW and DW of roots with increasing auxin concentrations (Figure 1). Among the auxins, IBA and NAA at a concentration of 2000 and 1000 ppm, respectively, significantly improved the FW of roots and shoots compared to other auxin treatments and the control (no exogenous auxins).

**Figure 2:**
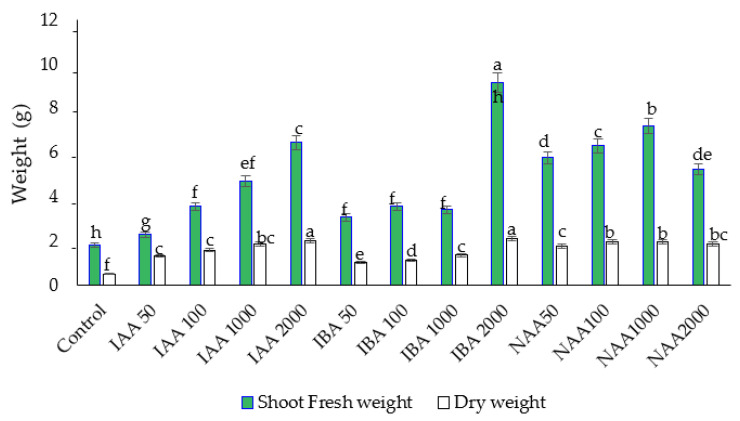
The effect of auxin concentration on shoot fresh and dry weight in *C. indicum.* Different letters indicate statistical differences as assessed by Duncan’s multiple comparison tests (*p* < 0.05).

Lower auxin concentrations had less influence on shoot FW and DW. An increase or decrease in NAA concentration from 1000 ppm decreased the FW and DW of roots and shoots. Maximum improvement in the FW and DW of shoots and roots was observed in the cutting treated with 2000 ppm IBA (Figure 2).

### 2.2. Effect of Temperature on Adventitious Root Number and Root Length in C. indicum

**Figure 3:**
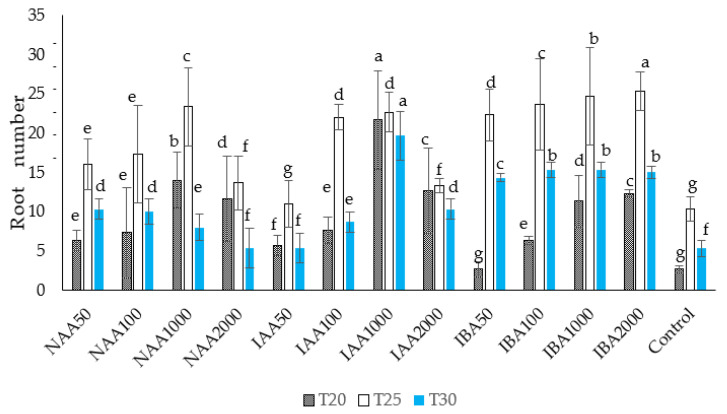
The effect of auxin concentration on average root number in *C. indicum.* Different letters indicate statistical differences as assessed by Duncan’s multiple comparison tests (*p* < 0.05).

In the present study, temperature appeared to be critical to adventitious root number and root length in *C. indicum* (Figure 3). The suitable temperature for rooting was 25 °C. When the cuttings were treated with IBA (2000 ppm) and grown at a soil temperature of 25 °C, they developed a higher number of adventitious roots than those grown at 20 °C or 30 °C. In the present study, a significantly greater number of adventitious roots were observed in the 2000 ppm IBA treatment followed by the IBA 1000 ppm treatment at 25 °C. The lowest number of roots was observed in the cuttings treated with IBA 50 ppm at 20 °C, which were not significantly different from the control (in the absence of auxin treatment) at *p* < 0.05. A significantly higher number of roots was also observed in the NAA and IAA treatments at concentrations of 1000 ppm at 25 °C. The number of roots recorded at a temperature of 30 °C was significantly lower compared to the root number at 25 °C.

**Figure 4:**
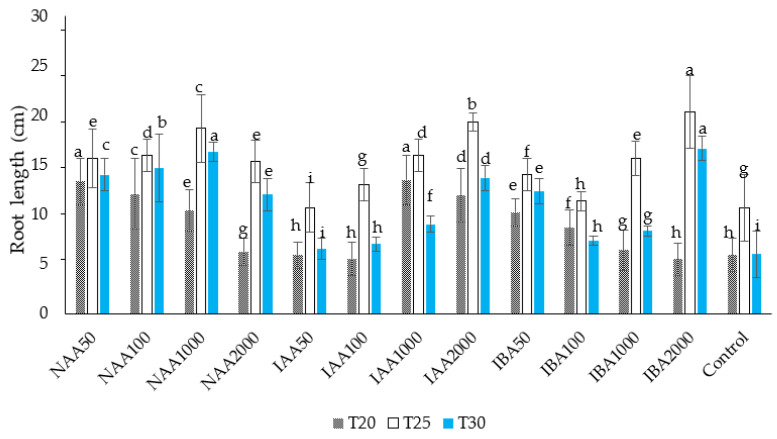
The effect of auxin concentration on an average root length in *C. indicum.* Different letters indicate statistical differences as assessed by Duncan’s multiple comparison tests (*p* < 0.05).

In the present study, a significantly greater root length was observed in the 2000 ppm IBA treatment at 25 °C. The lowest number of roots was observed in the cuttings treated with IBA 50 ppm, which were not significantly different from the control (in the absence of auxin treatment) at *p* < 0.05. A significantly higher root length was also observed in the IAA treatments at concentrations of 2000 ppm at 25 °C. Root length decreased significantly when the temperature of growth substrates was maintained at 20 °C and 30 °C compared to root length at 25 °C (Figure 4).

### 2.3. Influence of Explant Position and Soil Types on Root Length, Bud Length, and Bud Number

**Figure 5:**
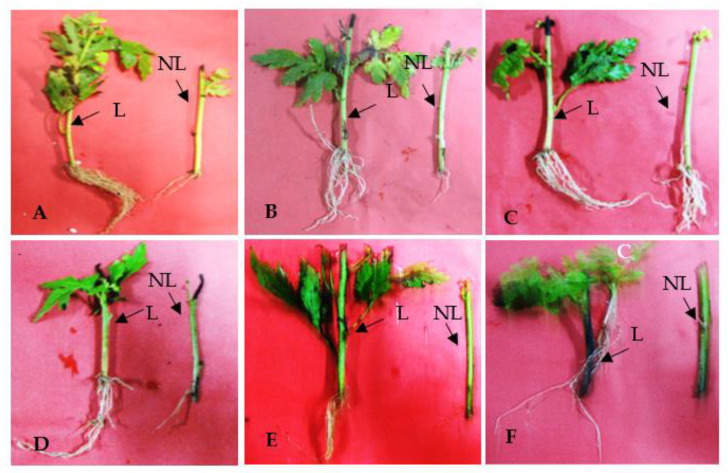
Rooting and sprouting of leafy and non-leafy stem cutting of *C. indicum* in the substrate containing vermiculite and perlite (1:1). (**A**–**C**) Cuttings treated with 2000 ppm of IBA. (**A**) Apical cutting, (**B**) median cutting, (**C**) basal cutting. (**D**–**F**) Cuttings in the absence of auxin treatment (control). (**D**) Apical cutting, (**B**) median cutting, (**C**) basal cutting. L: leafy, NL; non-leafy cutting.

In the present study, explant position (apical, median, and basal parts) significantly affected the different growth parameters of cuttings eight weeks after their establishment (Figure 5). Larger root lengths and root numbers were observed in the cutting explants obtained from the apical and basal regions, respectively (Table 1). The maximal number of buds was recorded in the cut obtained from the median portion of the stem. The average number of buds that emerged from the apical cuttings was significantly lower than that of the other types of cuttings, whereas their bud length was the highest. The position of the explant showed no distinct effect in terms of the number of leaves, especially between apical and basal explants. A similar trend was observed in the case of root length between median and basal explants.

The cuttings obtained from apical explants were basally treated with 2000 ppm IBA and grown in different types of growth substrates. In the present study, the growth medium significantly affected the different growth parameters of cuttings eight weeks after their establishment (Table 1). When we compared the type of growth substrate (vermiculite and perlite, Santro, and sandy loam soil) in the rooting medium of cuttings, the apical cuttings planted in the vermiculite and perlite (V + P) at a ratio of 1:1 and Santro showed better rooting parameters compared to sandy soil, indicating that cutting position and substrate type significantly influence root initiation and the rooting system, respectively. Other growing parameters, such as average root number and bud lengths, were higher in the cuttings grown in Santro. However, growth substrate type had no significant effect on leaf number in plants, especially between vermiculite and perlite (V + P) and Santro. Leafy stem cuttings had a higher average number of roots, root length, leaves number, bud length, and number of buds compared to leafless cuttings (Table 2).

### 2.4. Influence of Growth Regulators on Chlorophyll Content

**Figure 6:**
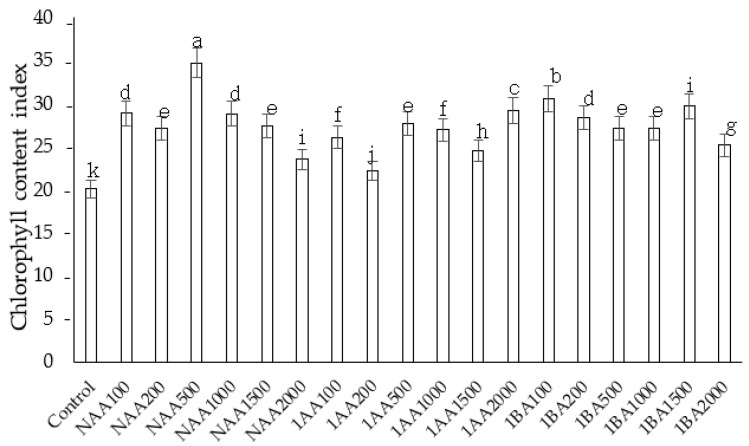
The effect of auxins on total chlorophyll content index in *C. indicum.* Values are reported as mean ± standard deviation of three parallel experiments. Different letters indicate statistical differences as assessed by Duncan’s multiple comparison tests (*p* < 0.05).

Cuttings showed a wide variation in total chlorophyll content (Figure 6). Chlorophyll levels increased in the auxins treated cuttings compared to control plants. However, there was no clear trend in the chlorophyll content of the treated leaves with increasing auxin concentration. Among all the treatments, higher levels of chlorophyll were observed in the cuttings treated with 50 ppm IBA. An IBA content higher than 50 ppm decreased the chlorophyll content of the cuttings. However, no significant difference was observed in the total chlorophyll content in cuttings treated with 50 ppm and 1000 NAA ppm.

### 2.5. Effect of Auxin Types and Concentration on Total Phenol Content (TPC) and Total Flavonoid Content (TFC)

**Figure 7:**
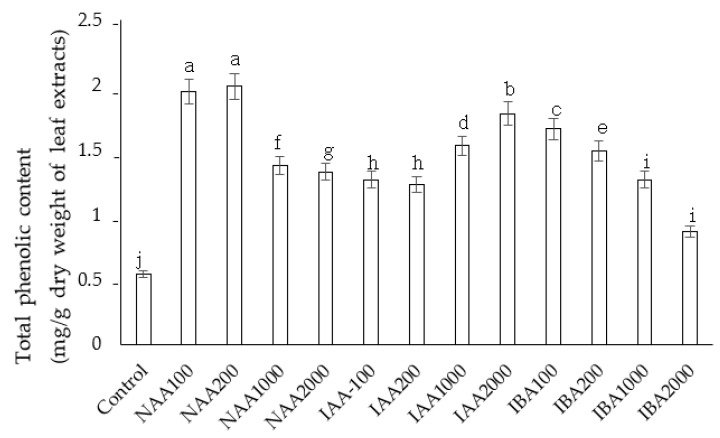
The effect of auxin concentration on total phenolic content in *C. indicum*. Values are reported as mean ± standard deviation of three parallel experiments. Different letters indicate statistical differences as assessed by Duncan’s multiple comparison tests (*p* < 0.05).

**Figure 8:**
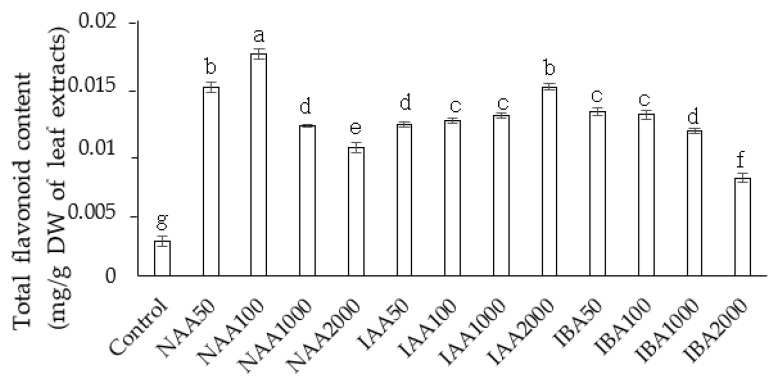
The effect of auxin concentration on total flavonoid content in *C. indicum.* Values are reported as mean ± standard deviation of three parallel experiments. Different letters indicate statistical differences as assessed by Duncan’s multiple comparison tests (*p* < 0.05).

TPC and TFC increased in the auxin-treated cuttings (Figure 7 and Figure 8). The TPC remained at the maximum value after treatment with 100 ppm NAA. A further increase or decrease in NAA lowered TPC compared to other auxin treatments and control plants. Higher IBA concentrations decreased TPC in a concentration-dependent manner. However, there was no clear trend in TPC content in the treated cuttings at different IAA concentrations.

TFC increased and peaked in the NAA treatment at a concentration of 100 ppm, while it significantly decreased at higher NAA concentrations. All the IAA concentrations increased the TFC in a concentration-dependent manner. Similarly, higher IBA concentrations produced a lower TFC in the cuttings in a concentration-dependent manner.

### 2.6. Phenolic Compounds

Protocatechuic acid, gentisic acid, chlorogenic acid, biochanin A, salicylic acid, caffeic acid, and glycitein were the most dominant phenolic compounds present in *C. indicum* (Figures S1 and S2). Except for IBA, lower auxin concentrations reduced the total phenolic compound content (Table S2 and Table 3). Phenolic compounds such as chlorogenic acid, biochanin, and salicylic acid were recorded higher in lower concentrations of IBA (50 PPM). Treatment with 2000 ppm IAA resulted in the highest elevation in total phenolic compounds (24,847.40 μg/g), whereas 100 ppm IAA showed the lowest total phenolic compound concentration (891.40 μg/g) relative to the control plants (6521.30 μg/g).

### 2.7. Principal Component Analysis (PCA) Root System Parameters in C. indicum.

**Figure 9:**
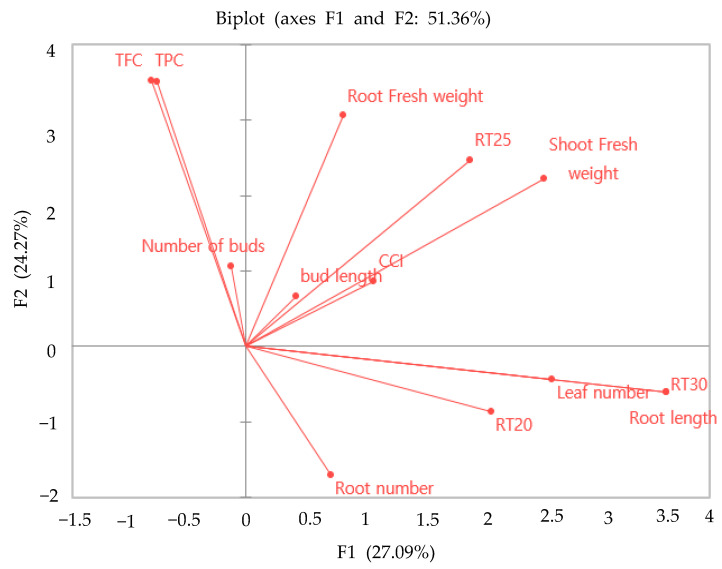
Principal component analysis results obtained for the qualitative morphological and physiological traits of *C. indicum*. RT20, room temperature of 20 °C; RT25, room temperature of 25 °C; RT30, room temperature of 30 °C; CCI, chlorophyll content index; TPC, total phenolic content; TFC, total flavonoid content.

PCA for 13 growth parameters was performed to visually interpret and examine the association between different growth parameters (Figure 9). The first two components explained approximately 52% of the variance. Principal component (PC) 1 accounted for 27.09% of the total variation and seemed to be associated with growth parameters, such as shoot FW, root FW, bud length, and chlorophyll content at 25 °C. PC2 accounted for 24.27% of the total variation and was characterized by a higher leaf number, root length, and root number at 20 °C and 30 °C.

## 3. Discussion

Adventitious root formation during cutting is often a limiting factor in vegetative propagation. Cutting before planting is an effective method for developing root systems to improve the root number and rooting percentage [54]. External and internal factors influence adventitious root formation in cuttings, among which auxin type and concentration play crucial roles in initiating this process [55,56]. In the present study, the cuttings’ average root length and root number were significantly affected by auxin concentration and type. Among the auxins tested, IBA was more effective than other tested auxins. Significantly longer adventitious roots were observed for the 2000 ppm IBA treatment. The lowest number of roots was observed in cuttings treated with 50 ppm IAA. The wide variation in the morphological response of cuttings to different auxins could be due to the chemical nature of auxins and the mode of action. However, in the case of IAA and NAA, higher auxin concentrations did not yield better rooting. Several previous studies have reported the beneficial and successful establishment of a rooting system of cuttings using auxins in different plant species [57,58]. However, in some cases, adding auxins at higher concentrations than required caused an inhibitory effect on adventitious root formation [59] and could cause callosity via excessive cell proliferation or inhibit root formation and shoot growth [60]. The present study showed that root induction from cuttings at various IAA and NAA concentrations increased up to the threshold level. Further increasing auxin concentration caused a decrease in the root formation and FW of the cuttings. Higher auxin levels have been observed to stimulate the biosynthesis of ethylene, triggering abscisic acid (ABA) synthesis in plants. Higher ABA concentrations cause stomatal closure, chloroplast damage, and ethylene production, leading to leaf senescence, necrosis, and ultimately plant death [61]. This indicates that growth regulators influence not only the rooting system but also photosynthetic parameters.

The stimulatory effect of auxins in producing adventitious roots in the cuttings varies from one species to another, or within the species, and differs largely due to the variations in the physiological, biological, and anatomical conditions [62]. This study observed a higher rooting percentage from cuttings treated with 2000 ppm IBA, which decreased in a concentration-dependent manner. In contrast, higher root formation was observed in *Stereospermum suaveolens* [63]. In a similar study, *Maytenus* sp. did not respond to the application of auxins such as IBA [64]. Higher IBA concentrations decreased the rooting percentage in *Syzygium cumini* [65]. Another report observed a very high rooting system in *Santalum album* [66], *Gmelina arborea* [67], and *Syzygium malaccense* [68] with and without IBA, indicating that IBA’s influence on root formation during cutting is species-specific and that different plant species require diverse IBA concentrations to induce higher root percentages. The increase in the number of roots per cutting due to IBA treatment found in this study corroborates previous findings [69]. In contrast, NAA more effectively induced more roots in other plant species [70]. However, applying auxins often does not promote the rooting percentage in some plant species [71,72]. Our results show that auxins significantly affected the rooting percentage and root length of cuttings and were critical in the rooting process. Moreover, it has been argued that applying auxins causes the release of energy and mobilization of proteins required for cell division and differentiation at the site of root primordia development [73]. Others have argued that exogenous auxin treatment stimulates the recruitment of carbohydrates in the shoot and increases the availability of sugars at the site of root primordia formation to act as a major carbon source. The sugars are utilized to produce the energy necessary for cell division and differentiation to trigger root initiation [74,75,76,77]. A similar tendency was also reported with rooting following auxin treatment [75,78]. Similarly, Hartmann et al. [62] observed that exogenously applying auxins activated vascular cambium cells and promoted adventitious root formation and stem cutting growth. Moreover, previous research indicated that auxin treatment influenced the accumulation of amino acids, such as aspartic acid, glycine, tyrosine, glutamic acid, and tryptophan, in *Ascophyllum* cuttings, which correlated strongly with rooting performance [79], which might be the case here. Although many others have successfully established adventitious roots in cuttings via auxin treatment in other plant species, the mechanisms triggering root formation and development by auxin treatment are contradictory [74,75,76,77]. It has been reported that a higher number of adventitious roots take up sufficient nutrients and water for the growth of cuttings and cause greater biomasses [80].

At the end of the eighth week, 100% rooting was observed regardless of treatment, indicating that chrysanthemums are easy to root. Among the three growth substrates tested, vermiculite and perlite at a ratio of 1:1 were more effective than Santro and soil in promoting the rooting of cuttings. Oh et al. [81] reported similar results, in which chrysanthemum sp. cuttings grown in perlite increased root numbers, root lengths, and dry weight. An increase in bud number and length can be attributed to increased root number and root length in the treated cuttings, as a higher number of roots absorb more water and nutrients from the growing substrate, leading to the production of more buds. This observation was further supported by the significant and positive correlation between bud number and the number of roots in the treated cuttings. The higher root formation in the perlite and vermiculite could be attributed to the higher water holding capacity and good aeration of the growing media. In contrast to the other substrate types, the aeration in the soil was low, which could have contributed to the lower root formation. Moreover, in a similar study, Khayyat et al. [82] observed that better aeration, drainage, and water holding capacity are important parameters for the improved root formation in *Epipremnum aureum* cuttings, indicating that rooting may be influenced by other factors, such as proper drainage and aeration properties of the substrate used for plant growth.

In the present study, the position of the cuttings collected from the donor plants influenced the rooting capacity. The average number of roots, root length, and biomass were higher in the apical segments than in the medium and basal segments. Similar results have been reported for the *Rosa hybrid* [83]. In contrast, another report observed no significant difference in rooting rate when the cuttings were collected from different positions along with the donor plants [84], indicating that the rooting potential of the cutting is significantly affected by the physiological state of the explants used in the experiment.

In the present study, leaves in cuttings significantly influenced the rooting system. The cuttings obtained from leafy nodes (LN) had a higher number of roots, root length, leaf number, bud length, and length, whereas no buds developed in cuttings from non-leafy nodes (NLN). In the present study, the average number of roots, root length, FW, and DW of the adventitious roots obtained from the LN apical cuttings were significantly higher than those of the basal and median cuttings. In contrast, Nicoloso et al. [85] reported that a higher root length was obtained from the median cuttings of *Hyptis suaveolens* (L.). In a similar study, Garbuio et al. [86] observed that the best root length was obtained for apical and median cuttings in *Pogostemon cablin* (Blanco) Benth. The mortality rate of basal cuttings without leafy nodes was superior to that of NLN apical cuttings, as per Amaro et al.’s [87] and Chagas et al.’s results [88]. The present study’s findings indicate that leaves in cuttings contribute to a better rooting system. Moreover, Pearson’s correlation analysis showed a significant and positive correlation between the number of leaves and root number. A significant positive correlation was also observed between leaf number and shoot FW (Table S1). The FW obtained from the apical LN cuttings showed greater FW and DW than NLN apical cuttings, probably due to the presence of leaves from the beginning of the experiments. It has been argued that the presence of leaves greatly affects bud growth by cuttings due to photoassimilate production, which is essential for bud formation [89]. Moreover, the young leaves are believed to be the major sources of auxins, after which they are transported basipetally to the stem in a sufficient amount to stimulate the root growth [90]

In the present study, the temperature at which the chrysanthemums grew was critical for adventitious root number and length. When the cuttings were treated with IBA (2000 ppm) and grown at a soil temperature of 25 °C, the cutting showed improved and faster rooting than the cutting grown at other temperatures (20 °C and 30 °C). Several previous studies have reported a close relationship between the temperatures of the stock plants before harvesting cuttings [91]. Moreover, the temperature of the growth medium influenced bud activity, growth rates, and flowering [92]. It has been reported that low temperatures during the growth of cuttings inhibit the activity of endogenous auxins [93]. Other studies have shown that reducing the temperature from 25 °C reduced the basipetal transport of auxins, such as IAA [94]. Thus, this could be a possible reason that the present study observed the lower root number, root length, number of buds, and bud length in chrysanthemum cuttings grown at 20 °C. These results align with Hansen et al.’s findings [95] for *Stephanotis floribunda,* which show reduced bud formation at a lower temperature (17 °C). It has been reported that applying IBA at higher temperatures increased cell wall plasticity more readily by activating ATPase located in the cell membranes [96]. Moreover, the PCA results indicate that the auxin treatment distinctly influenced the chlorophyll content and its association with rooting performance. It has been reported that chlorophyll content and photosynthesis rate are related to carbohydrate metabolism and energy production in plants, which are important for rhizogenesis at the initial rooting stage [97]. Others argue that the increase in the chlorophyll level in the cuttings may be a defense response to stress tolerance during unfavorable growing conditions at the initial rooting stage [98].

Several previous studies have reported the possible role of phenolic compounds in triggering primary root formation [99]. Moreover, it has been reported that phenolic compounds present in cuttings’ explants can influence metabolic processes, including the respiration rate, and protect auxins against oxidation by inhibiting the activity of IAA oxidase and phenol–oxidase complexes [100]. It is further argued that these complexes are cofactors for root primordia formation [100]. Thus, the influence of phenolics on peroxidase and polyphenol oxidase may influence the formation of root primordia in cuttings [96]. Other studies have observed that polyphenolic compounds can affect the activity of some enzymes that participate in rhizogenesis in cuttings [101]. They act as antioxidants to protect auxins from oxidation and plant tissues at the site of wounds from oxidative stress at the cutting site and promote a higher rooting percentage [101]. In this report, the high-performance liquid chromatography (HPLC) analysis of the auxin-treated chrysanthemums showed an increase in the phenolic compounds, which are important in the initial stage of adventitious root formation. In particular, the content of protocatechuic acid, salicylic acid, and gentisic acid significantly increased in all the auxin treatments. Similarly, a higher auxin concentration increased the accumulation of chlorogenic acid relative to the control plants. Previous studies have shown that phenolic compounds, such as chlorogenic acid, caffeic acid, and gallic acid, impact rooting cofactors and protect IAA against oxidation [102]. Other studies have reported that phenolic compounds (chlorogenic acid, epicatechin, caffeic acid, catechol, gallic acid, and ferulic acid) are critical during the initial root formation stage by protecting auxins (IAA) against oxidation [103,104] or function as free radical scavengers and increase the auxin concentration to trigger adventitious root formation [104,105]. Therefore, based on the data obtained in the present study, it can be assumed that the enhanced accumulation of phenolic compounds in the cuttings may have enhanced cell division and differentiation into root primordia regulated by auxins.

## 4. Materials and Methods

### 4.1. Chemicals

All chemicals used in this study were of analytical grade. Compounds, such as the Folin–Ciocalteu reagent, quercetin, tert-butyl-4-hydroxytoluene (BHT), 2,2-diphenyl-1-picryl-hydrazyl-hydrate (DPPH), 2,20-Azino-bis(3-ethylbenzothiazoline-6-sulfonic acid) (ABTS), Trolox, gallic acid, IBA, IAA, and NAA, were obtained from Sigma-Aldrich Chemical Co. (St. Louis, MO, USA). Ultrapure distilled water used in the analysis was purchased from the Zeneer Power 1 System (Human Corporation, Seoul, Korea). Standard phenolic compounds were obtained from Sigma Chemical Co. (St. Louis, MO, USA).

### 4.2. Experimental Site and Plant Material

The plant material was grown in a field at Kangwon National University experimental farm, at 37°5624.63 (N) latitude and 127°46 (E) longitude and 117.22 m above sea level. Samples were taken in September (2015 and 2016) from a very mature *C. indicum* plant. Perforated polythene bags were used for collection from the growing field, and the cuttings were used within 1 h of collection. The voucher specimens were deposited in the Herbarium, Department of Biological Sciences, College of Natural Sciences, Kangwon National University, Chuncheon, South Korea.

### 4.3. Stem Cutting Preparation and Applying Growth Regulator

Cuttings were collected during the early morning (from 9:00 to 10:00 am) and maintained at moist and cool temperatures using perforated polyethylene bags during their transportation from the experimental field to the greenhouse. Cuttings were prepared with an average length of 15.50 ± 0.65 cm and diameter of 0.89 ± 0.23 mm, discarding the apical 2–3 cm. The cuttings were treated with various freshly prepared concentrations (50 ppm, 100 ppm, 1000 ppm, and 2000 ppm) of IAA, IBA, and NAA, as described by Kroin [106], by dipping their basal (2.5 cm) portions in various test solutions for 24 h at room temperature (25 °C). An untreated set of cuttings was dipped in water as a control. The apical cut ends of each cutting were covered with paraffin wax to minimize water loss. Each treatment consisted of 20 cuttings. After the growth regulator treatment, cuttings were dried in cold air for 1–2 min before being placed in growing media. Every treated and untreated cutting was planted in polybags (12 cm × 12 cm) containing sterilized rooting medium, viz. vermiculite and perlite (1:1), Santro (organic potting mix, Seoul Bio Co., Ltd., Seoul, South Korea), and sandy loam soil (pH 6.75, organic matter 9.30%, and phosphorus 1.20%). The cuttings were planted vertically in polybags of 800 mL of the volume of rooting medium (one cut in each bag). The planted cuttings were shifted to the growth chamber at 20 °C, 25 °C, and 30 °C, 50–60 relative humidity, with a photoperiod of 16 h (~500 μmol·m^−2^·s^−1^ irradiance) and darkness for 8 h. The root and bud initiation of the cuttings was first observed after approximately two weeks. After collecting the morphological traits of the cuttings, these plantlets were re-planted in polybags containing a growing medium.

### 4.4. Experimental Design

The cuttings were watered regularly. The experiment was designed using a randomized complete block design (RCBD). This experiment was performed in triplicate for three seasons. After eight weeks, the mature stem cuttings were recorded for the shoot and root traits: (1) number of nodes, (2) the maximum number of primary shoots, (3) shoot length, (4) number of cuttings that showed rooting, (5) the maximum number of adventitious roots, (6) bud length and number, (7) fresh weight and dry weight of roots and shoots, and (8) root length.

#### 4.4.1. Sample Collection and Extract Preparation

The fresh and fully developed roots (20 g) were collected and thoroughly washed with distilled water and freeze-dried for one day. Approximately 2 g of the finely ground samples was mixed with 20 mL of 80% methanol at room temperature (25 °C). Then, the mixture samples were filtered through filter paper (Whatman No. 1) and concentrated at 40 °C in a rotary evaporator (Eyela, SB-1300, Shanghai Eyela Co., Ltd., Shanghai, China). The obtained residue was suspended in 10 mL of methanol (80%). The extractions were performed in triplicate for each root sample and used for further analysis.

#### 4.4.2. Determining Total Phenolic Acid Content (TPC)

Total phenolics were extracted using the Folin–Ciocalteu assay, following the method described by Singleton et al. [107] with some modifications. Each extract (100 μL, 1 mg/mL) was added to a test tube containing a 50 μL of phenol reagent (1 M). The volume was increased by adding 1.85 mL of distilled deionized water, and the solution was allowed to stand for 3 min for a reaction after vortexing. After 3 min, 300 μL of Na_2_CO_3_ (20% in water, *v*/*v*) was added, and the final volume (4 mL) was adjusted by adding 1.7 mL of distilled deionized water. Reagent blanks were prepared using deionized distilled water. The final mixture was vortexed and incubated for 1 h in the dark at room temperature. The absorbance was measured at 725 nm using a Jasco V 530 UV-VIS spectrophotometer. The standard curve was prepared using 0 mg L^−1^, 65.5 mg L^−1^, 125 mg L^−1^, and 250 mg L^−1^ solutions of gallic acid in methanol: water (50:50, *v*/*v*). Total phenol values are expressed in terms of the gallic acid equivalent (GAE) of the plant’s DW. All determinations were performed in triplicate.

#### 4.4.3. Determining Total Flavonoid Content (TFC)

Adventitious roots cultured in a liquid medium supplemented with various sucrose concentrations were used to determine TFC. The TFC in the extracts was determined as described by Moreno et al. [108]. A 0.5 mL sample (1 mg/mL) was mixed with 0.1 mL of 10% aluminum nitrate and 0.1 mL of potassium acetate (1 M). Then, 4.3 mL of 80% ethanol was added to the mixture to reach a total volume of 5 mL. The mixture was vortexed, and the solution was allowed to stand for 40 min at room temperature. The absorbance was measured spectrophotometrically at 415 nm. All determinations were performed in triplicate. Total flavonoid values are expressed in terms of the quercetin equivalent (Qu) of the plant’s DW. The standard curve was prepared using 0 mg L^−1^, 5 mg L^−1^, 10 mg L^−1^, and 100 mg L^−1^ quercetin solutions.

#### 4.4.4. Estimation of Phenolic Compounds

The phenolic compound content in *C. indicum* was estimated by liquid chromatography–mass spectrometry (LC-MS/MS), as described previously by Ghimire et al. [109]. An Agilent 1200 Series HPLC system (Agilent 1200, Agilent Technologies, Palo Alto, CA, USA) was equipped with a pump, degasser, autosampler, and column (Agilent 1100 series, Agilent Technologies, Palo Alto, CA, USA) coupled to a mass spectrometer (Applied Biosystems, Beamsville, ON, Canada). The negative ion mode was used. The parameters used to determine the phenolic compounds present in the samples were as follows: nebulizer gas pressure, 40 psi; drying gas pressure, 70 psi; collision gas pressure, 2 psi; and curtain gas pressure, 20 psi. The drying gas temperature and capillary voltage were set to 350 °C and 4.5 kV, respectively. The mobile phase consisted of 0.1% HCOOH (*v*/*v*) in water (mobile phase A) and 0.1% C_2_H_3_N in water (95:5, *v*/*v*). The flow rate was 0.7 mL min−1, and the gradient was as follows: 10–40% B for 0–10 min; 40–50% B for 10–20 min; 50–100% B for 20–25 min; 100–10% B for 25–26 min; and 10% B for 26–30 min. The phenolic compounds were separated using a C18 column (4.6 mm × 250 mm, 5 µm). The compounds were then separated at 25 °C. The injection volume was 10 µL. The process was carried out in negative mode in the multiple reaction monitoring (MRM) mode. An electrospray ion source (ESI) recorded the mass spectrometry data. Different mass spectrometric parameters, such as entrance potential (EP), collision energy (CE), declustering potential (DP), cell entrance potential (CEP), and collision cell exit potential (CXP), were determined for each MRM transition monitored.

#### 4.4.5. Statistical Processing

The data are presented as the mean ± standard deviation values of independent replications. Statistical processing related to the test results was analyzed by ANOVA using the SPSS program (Statistical Package for Social Science, Version 24). Significant differences at *p* < 0.05 between the control and experimental groups were verified by Duncan’s multiple range test.

## 5. Conclusions

This is the first systematic study to present results for the regeneration of *C. indicum* cuttings and provide useful information about the biochemical and physiological parameters involved during the propagation process. It has been shown that exogenously applying auxins can stimulate root formation in *C. indicum*, and that vegetative propagation of *C. indicum* via cutting is viable. The results recommend using 2000 ppm IBA to achieve more effective adventitious root regeneration than other auxins. Overall, rooting was best in the apical cuttings portion of the stem grown in the vermiculite and perlite substrate at a ratio of 1:1. The elevated number of phenolic compounds in the treated cuttings suggested that these phytochemicals are involved in the tissue’s physiological state and adventitious root formation. Improved propagation methods could provide opportunities for massive production and increase the number of planting materials for commercial applications.

## Figures and Tables

**Table 1:** The effect of stem cutting position and growth substrates on root number, root length, leaf number, bud length, and bud number in *C. indicum*.

Characteristics		Number of Roots	Root Length (cm)	Number of Leaves	Number of Buds	Bud Length (cm)
Stem cutting position	Apical	9.33 ± 0.47 ^c^	19.33 ± 1.25 ^a^	10.00 ± 1.63 ^a^	2.33 ± 0.47 ^c^	8.66 ± 0.94 ^b^
	Median	9.00 ± 0.81 ^c^	16.00 ± 1.63 ^b^	8.33 ± 1.24 ^b^	5.33 ± 0.47 ^a^	4.33 ± 1.24 ^c^
	Basal	11.00 ± 0.82 ^b^	16.00 ± 0.82 ^b^	10.33 ± 1.25 ^a^	4.00 ± 0.82 ^b^	1.27 ± 0.17 ^d^
Substrates	V + P	9.33 ± 0.47 ^c^	18.6 ± 1.63 ^a^	6.97 ± 2.49 ^c^	4.50 ± 1.63 ^b^	4.67 ± 2.49 ^c^
	Santro	13.00 ± 2.16 ^a^	15.00 ± 0.82 ^b^	6.67 ± 1.89 ^c^	2.33 ± 0.47 ^c^	13.33 ± 3.39 ^a^
	Sandy Loam Soil	5.00 ± 1.10 ^d^	8.00 ± 1.20 ^c^	8.00 ± 1.50 ^b^	4.00 ± 0.06 ^b^	2.00 ± 0.02 ^d^

Within a column, the mean followed by the same letter did not differ significantly according to Duncan’s multiple comparison tests (*p* < 0.05).

**Table 2:** The effect of leafy and non-leafy explants on root number, root length, leaf number, bud length, and bud number in *C. indicum*.

Characteristics	Number of Root	Root Length (cm)	Number of Leaves	Number of Buds	Bud Length (cm)
Leafy cutting	12.00 ± 1.63 ^a^	20.00 ± 1.63 ^a^	6.67 ± 0.94 ^a^	3.00 ± 0.82 ^a^	8.00 ± 0.82 ^a^
Non-leafy cutting	9.50 ± 0.81 ^b^	18.00 ± 0.81 ^b^	5.33 ± 1.25 ^b^	2.67 ± 0.47 ^b^	2.33 ± 0.47 ^b^

Within a column, the mean followed by the same letter did not differ significantly according to Duncan’s multiple comparison tests (*p* < 0.05).

**Table 3:** Distribution of total phenolic compounds in *C. indicum* treated with different concentrations of auxins.

Treatment	GA	Pro	Gen	*p*-Hy	Chl	*p*-C	FA	Bio	Hom	Sal	Van	CA	Vit	Gly	Api	Lut	L-Phe	TPC
	(μg/g)																	
Control	LOD	162.2	9.2	26.1	5763	LOQ	ND	371.0	LOD	131.8	ND	57.7	ND	LOQ	ND	LOQ	LOD	6521
NAA50	LOQ	525.0	24.7	LOD	336	ND	ND	137.3	LOQ	385.0	ND	119.7	ND	355.3	91.2	19.8	LOD	1994
NAA 100	LOQ	464.0	28.2	LOD	2672	ND	ND	13.4	LOD	626.0	ND	53.2	ND	LOQ	ND	57.2	LOD	3914
NAA 1000	LOD	375.4	18.8	ND	19,691	ND	ND	87.5	LOQ	287.6	ND	78.6	ND	LOQ	ND	74.9	ND	20,614
NAA 2000	LOQ	338.3	16.8	LOQ	18,223	ND	ND	975.3	LOQ	278.8	ND	11.3	ND	LOQ	LOD	46.4	LOD	19,890
1AA50	LOQ	219.6	21.2	55.0	186	LOQ	ND	84.7	ND	246.5	ND	77.7	ND	LOQ	ND	LOD	LOQ	891
1AA100	LOQ	337.3	28.4	89.5	134	ND	ND	178.7	LOQ	614.7	LOD	47.3	ND	LOQ	27.7	37.2	LOQ	1494
1AA1000	LOD	375.3	23.6	74.1	20,095	LOQ	ND	544.7	LOD	443.3	ND	19.0	ND	LOQ	73.6	19.0	LOD	21,667
1AA2000	LOD	498.8	25.9	77.0	22,715	LOQ	ND	1288.0	LOD	149.5	ND	134.9	ND	LOQ	LOQ	58.3	LOQ	24,947
1BA50	75.3	175.5	23.4	LOQ	2678	ND	ND	1364.8	LOQ	459.2	ND	12.1	LOQ	LOQ	153.2	31.3	ND	4973
1BA100	LOQ	233.3	15.8	LOQ	5566	ND	ND	329.6	LOQ	195.4	ND	29.7	ND	LOQ	ND	15.4	LOD	6385
1BA1000	ND	234.0	19.7	LOQ	1256	ND	ND	487.3	LOD	293.3	ND	68.6	ND	LOQ	ND	99.5	LOQ	2459
1BA2000	LOD	183.3	15.3	LOQ	9515	LOQ	ND	852.5	LOQ	426.3	ND	751.5	ND	185.2	ND	LOQ	LOQ	11,229

Abbreviation: GA, gallic acid; Pro, protocatechuic acid; Gen, gentisic acid; *p*-Hy, p-Hydroxybenzoic acid; Chl, chlorogenic acid; *p*-C, p-coumaric acid; FA, ferulic acid; Bio, biochanin; Hom, homogentisic acid; Sal, salicylic acid; VA, vanillic acid; CA, caffeic acid; Vit, vitexin; Gly, glycitein; Api, apigenin; Lut, luteolin; L-Phe, L-Phenylalanine; TPC, total phenolic compounds; ND, not detected; LOD, limit of detection; LOQ, limit of quantification.

## Data Availability

Not applied.

## Supplementary Materials

The following supporting information can be downloaded at: https://www.mdpi.com/article/10.3390/plants11111440/s1. Table S1. Pearson’s correlation coefficient among growth parameters. Table S2. Calibration curves of the equation of 33 phenolic compound standards. Figure S1. MRM ion chromatogram of the selected 33 phenolic compound standards. Figure S2. Representative MRM ion chromatogram of phenolic compounds from root sample treated with 2000 ppm of IBA.
