# Supplementary material for: Biochemical and Physiological Changes during Early Adventitious Root Formation in Chrysanthemum indicum Linné Cuttings
Source: Plants (Basel). 2022 May 28;11(11):1440. doi: 10.3390/plants11111440 (PMC9183066; doi:10.3390/plants11111440)
Supplement: Supplementary file 1 [file plants-11-01440-s001.zip › plants-1674168-supplementary.pdf]

Table S1. Pearson's correlation coefficient among growth parameters.

* Variables	Root Fresh weight	Shoot Fresh weight	Root length	Root number	Number of buds	Bud length	Leaf number	TPC	TFC	Chl
Root Fresh weight	**1**									
Shoot Fresh weight	0.630	**1**								
Root length	0.166	0.511	**1**							
Root number	-0.443	-0.092	0.082	**1**						
No of buds	0.002	0.087	-0.114	-0.042	**1**					
bud length	-0.227	0.058	-0.029	0.211	0.148	**1**				
Leaf no	-0.079	0.195	0.670	0.428	0.151	0.187	**1**			
TPC	0.414	0.200	-0.323	-0.282	0.350	0.194	-0.096	**1**		
TFC	0.466	0.228	-0.336	-0.263	0.137	0.119	-0.187	0.953	**1**	
Chl	0.179	0.184	0.107	0.246	-0.079	0.143	0.295	0.244	0.316	**1**

* TPC, total phenolic content; TFC. Total flavonoid content; Chl, Chlorophyll content index

Table S2. Calibration curves of the equation of 33 phenolic compound standards.

Compound	Conc. (μg/ml)	* LOD (ppm)	LOQ (ppm)	Linearity (r^2)	Equation	DP	EP	CEP	CE	CXP
5-Sulfosalicylic acid	0.5	0.04	0.15	0.999	y=342000x-33500	-21.0	-7.50	-10.0	-18.0	-44.0
Gallic acid	0.5	0.11	0.37	0.995	y=33900x+4440	-1.0	-7.000	-10.000	-14.000	-26.000
L-Phenylalanine	0.5	7.32	24.40	0.995	y=484x+2490	-51.0	-10	-12.62	-16	-30
Homogentisic acid	0.5	0.08	0.28	0.996	y=72800x-1550	-21.0	-7.0	-14.0	-14.0	-28.0
Catechin	0.5	0.38	1.25	0.997	y=11200x+2310	-81.0	-9.000	-12.000	-36.000	-20.000
Daidzin	0.5	0.06	0.18	0.995	y=27100x+17700	-66.0	-10.000	-28.000	-38.000	-46.000
Protocatechuic acid	0.5	0.36	1.19	0.991	y=15900x-6370	-31.0	-9.0	-8.0	-18.0	-22.0
Chlorogenic acid	0.5	0.01	0.02	0.998	y=378000x+54400	-16.0	-9.5	-18.0	-18.0	-36.0
Orientin	0.5	0.04	0.14	0.998	y=-5070x^2+71900x+4000	-61.0	-11.0	-20.0	-22.0	-54.0
Vanillic acid	0.5	0.91	3.03	0.994	y=3320x+2150	-61.0	-11.000	-20.000	-22.000	-54.000
Rutin	0.5	0.03	0.08	0.994	y=77000x+53200	-96.0	-8.0	-26.0	-48.0	-52.0
p-Hydroxybenzoic acid	0.5	0.09	0.29	0.998	y=45500x+14400	-16.0	-9.5	-14.0	-18.0	-18.0
Vitexin	0.5	0.03	0.09	0.994	y=-340x^2+45900x+22300	-56.0	-9.0	-28.0	-22.0	-50.0
Caffeic acid	0.5	0.01	0.03	0.993	y=282000x+62800	-11.0	-10.0	-12.0	-20.0	-30.
Naringin	0.5	0.04	0.12	0.995	y=47300x+14000	-126.0	-10.0	-28.0	-52.0	-32.0
Genistin	0.5	0.17	0.58	0.997	y=-140x^2+11300x+16100	-76.0	-11.0	-16.0	-42.0	-52.0
p-Coumaric acid	0.5	0.03	0.11	0.993	y=63500x+16800	-16.0	-5.0	-8.0	-20.0	-22.0
Ferulic acid	0.5	0.45	1.52	0.993	y=10200x+73.4	-6.0	-6.5	-12.0	-22.0	-26.0
m-Coumaric acid	0.5	0.03	0.11	0.999	y=6620x^2+74200x+39300	-21.0	-10.0	-18.0	-18.0	-26.0
Veratric acid	0.5	8.30	27.80	0.997	y=2080x-12200	-26.0	-8.0	-8.0	-16.0	-28.0
Gentisic acid	0.5	0.02	0.07	0.999	y=388000x-45900	-11.0	-8.0	-6.0	-28.0	-22.0
Myricetin	0.5	0.07	0.23	0.999	y=75200x+1160	-61.0	-10.0	-16.0	-36.0	-28.0
Luteolin	0.5	0.08	0.26	0.997	y=-020x^2+56100x+13600	-66.0	-10.0	-16.0	-46.0	-30.0
Quercetin	0.5	0.05	0.15	0.999	y=160000x+6730	-31.0	-10.5	-18.0	-28.0	-32.0
Apigenin	0.5	0.04	0.14	0.998	y=76700x+42400	-56.0	-8.5	-16.0	-48.0	-22.0
Naringenin	0.5	0.38	1.25	0.999	y=245x^2+13300x+6760	-66.0	-8.0	-16.0	-30.0	-26.0
Genistein	0.5	0.11	0.36	0.995	y=29400x+9740	-46.0	-8.0	-22.0	-38.0	-30.0

kaempferol	0.5	0.30	0.99	0.999	y=13300x+17700	-81.0	-8.5	-20.0	-72.0	-12.0
Hesperetin	0.5	0.04	0.14	0.997	y=93800x+45300	-51.0	-7.5	-20.0	-32.0	-32.0
Formononetin	0.5	0.01	0.02	0.999	y=274000x+21300	-21.0	-7.5	-14.0	-24.0	-44.0
Glycitein	0.5	0.60	2.00	0.998	y=28000x-25800	-11.0	-5.0	-14.0	-26.0	-42.0
Salicylic acid	0.5	0.02	0.07	0.997	y=606000x-45.2	-16.0	-6.0	-12.0	-20.0	-20.0
Biochanin A	0.5	0.03	0.09	0.997	y=113000x+49600	-46.0	-8.0	-14.0	-24.0	-48.0

* LOD, limit of detection; LOQ, limit of quantification; EP, entrance potential; CE, collision energy; DP, declustering potential; CEP, cell entrance potential; CXP, collision cell exit potential.

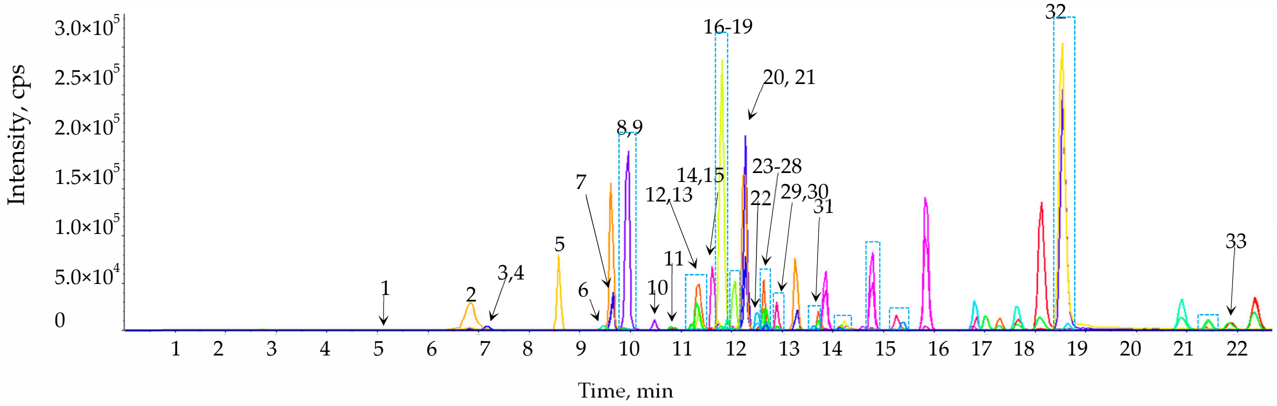

Figure S1. MRM ion chromatogram of the selected 33 phenolic compound standards.
1. 5-sulfosalicylic acid; 2. gallic acid; 3. L-phenylalanine; 4. homogentisic acid; 5. catechin; 6. daidzin; 7. protocatechuic acid; 8. chlorogenic acid; 9. orientin; 10. rutin; 11. *p*-hydroxybenzoic acid; 12. vitexin; 13. vanillic acid; 14. caffeic acid; 15. naringin; 16. genistin; 17. *p*-coumaric acid; 18. ferulic acid; 19. *m*-coumaric acid; 20. veratric acid; 21. gentisic acid; 22. myricetin; 23. luteolin; 24. quercetin; 25. apigenin; 26. naringenin; 27: Genistein 28. kaempferol; 29. hesperetin; 30. formononetin; 31. glycitein; 32. salicylic acid; 33. biochanin A.

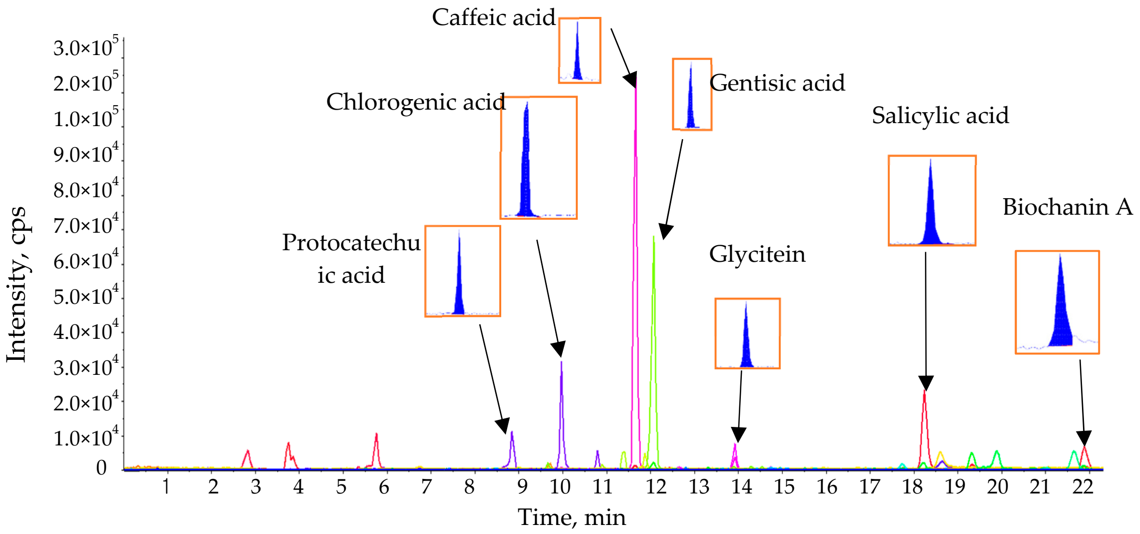

Figure S2. Representative MRM ion chromatogram of phenolic compounds from root sample treated with 2000 ppm of IBA.
